# Covered Stent Treatment of a Saphenous Vein Graft Pseudoaneurysm: A Rare Late Complication of CABG

**DOI:** 10.1016/j.jaccas.2026.109559

**Published:** 2026-07-30

**Authors:** Nisha S. Kolhe, Fatima Tooba, Jordan Sexe, Robert Smith, Gloria Elizondo

**Affiliations:** University of Texas Health Science Center at Tyler, Tyler, Texas, USA

**Keywords:** complication, computed tomography, coronary artery bypass, intravascular ultrasound, percutaneous coronary intervention, stents

## Abstract

**Background:**

Saphenous vein graft (SVG) aneurysms are a rare but potentially life-threatening complication after coronary artery bypass grafting, with an estimated incidence of 0.07%. Presentations range from incidental imaging findings to severe complications, including rupture, myocardial infarction, or compression of mediastinal structures.

**Case Summary:**

A 65-year-old man with prior 3-vessel coronary artery bypass grafting presented with progressively worsening substernal chest pressure. Coronary angiography and computed tomography angiography identified a 71 × 45-mm saccular pseudoaneurysm of the SVG. Following heart team discussion, the patient underwent intravascular ultrasound–guided percutaneous coronary intervention with sequential covered stent deployment, resulting in complete exclusion of the pseudoaneurysm.

**Discussion:**

Optimal management remains uncertain because of limited clinical data. This case emphasizes the feasibility of intravascular ultrasound–guided covered stent placement; to our knowledge, this is the largest reported de novo SVG pseudoaneurysm treated with this approach.

**Take-Home Message:**

In selected patients, covered stent placement offers a safe, minimally invasive alternative to surgery while preserving graft patency.

## Visual Summary

**Figure:**
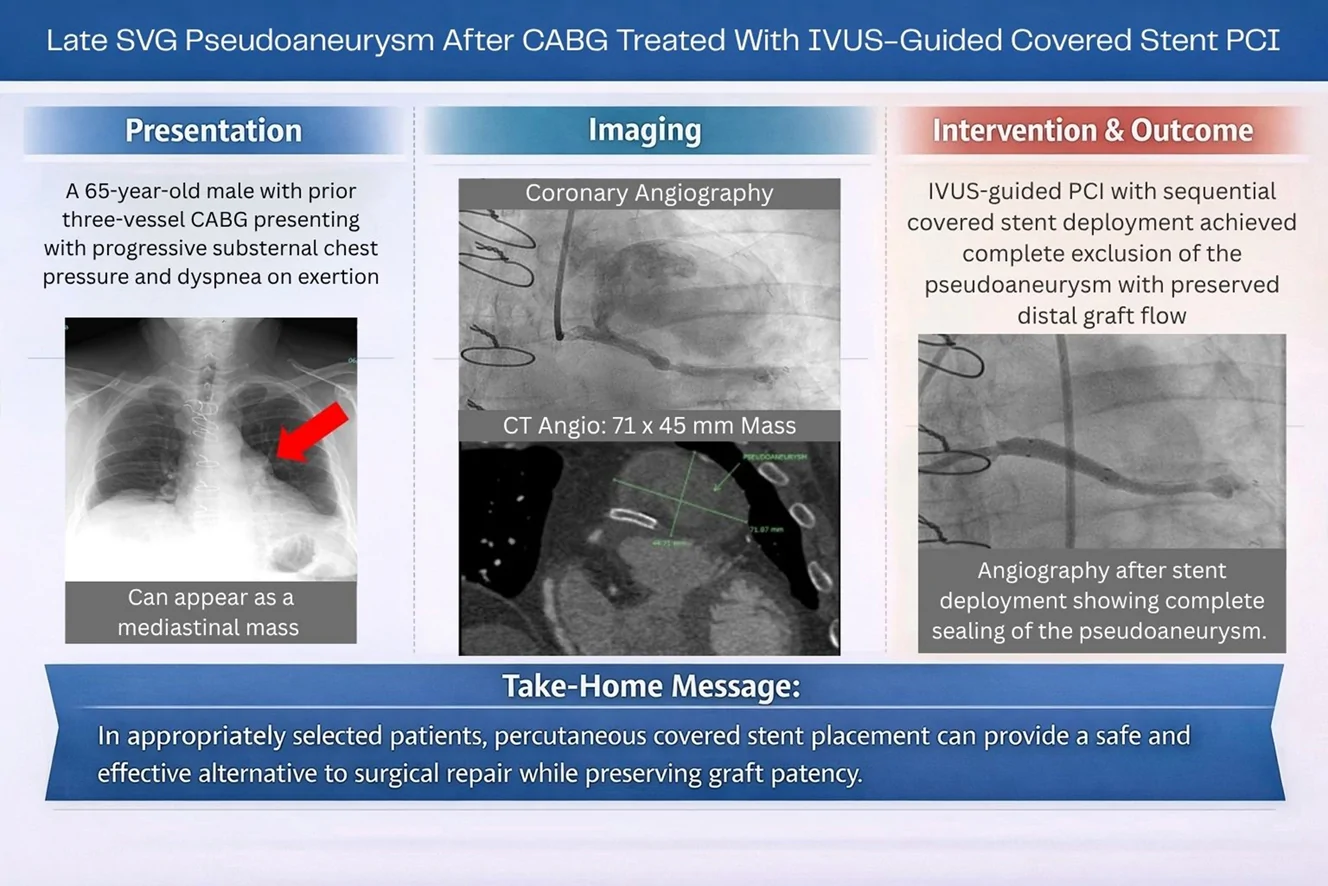

**Visual Summary:**
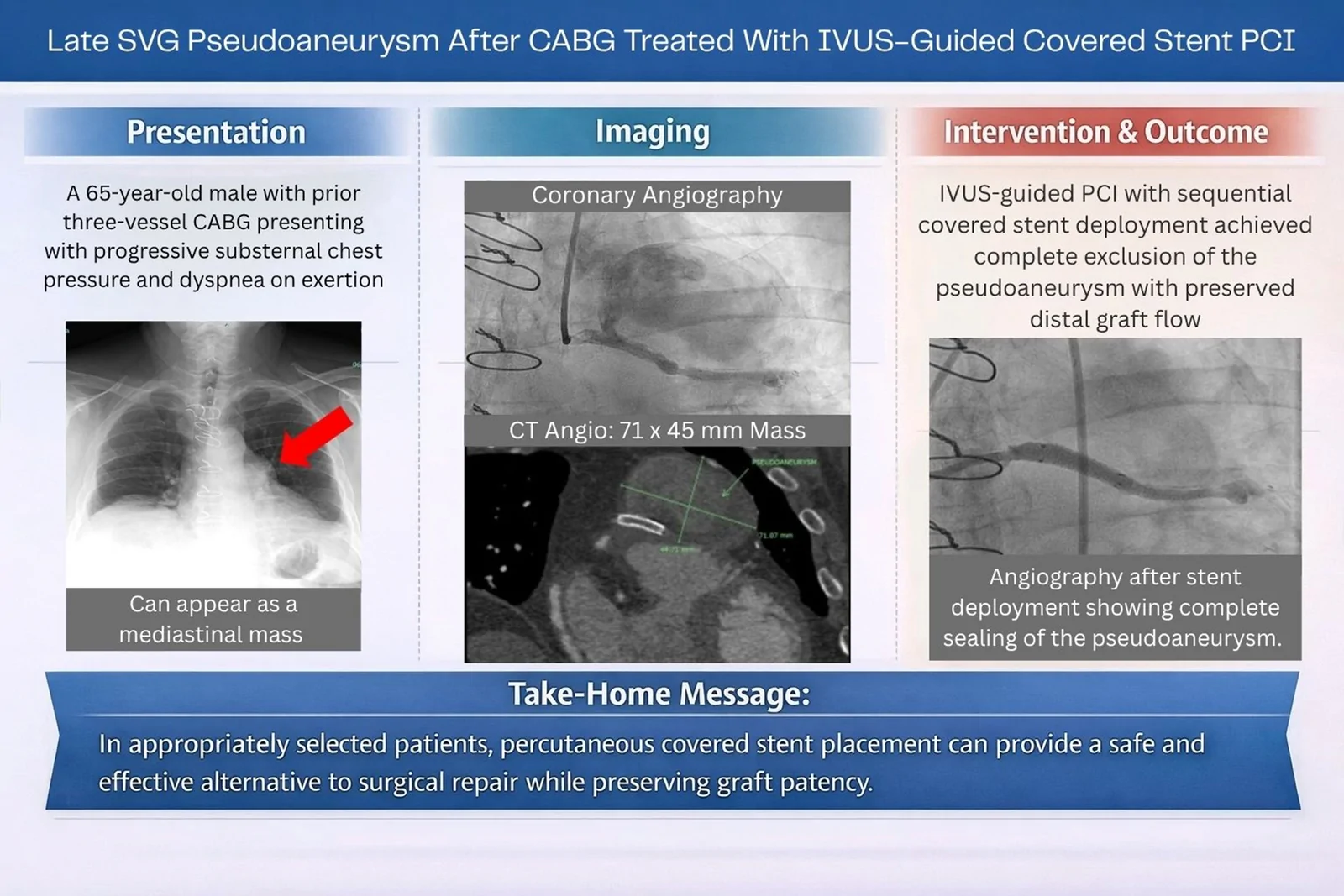
Diagnosis and Percutaneous Management of a Late Saphenous Vein Graft Pseudoaneurysm CABG = coronary artery bypass graft; CT Angio = computed tomography angiography; IVUS = intravascular ultrasound; PCI = percutaneous coronary intervention.

## History of Presentation

A 65-year-old man presented with progressively worsening substernal chest pressure without radiation over 18 months. The symptoms occurred with minimal exertion, accompanied by dyspnea, and were partially relieved with rest and antacids. He reported that dyspnea on exertion had historically represented his anginal equivalent.

**Take-Home Messages**

• Saphenous vein graft pseudoaneurysms are rare but potentially serious late complications of CABG that require prompt recognition and multimodality imaging for accurate diagnosis.
• In appropriately selected patients, IVUS-guided percutaneous covered stent placement can provide a safe and effective alternative to surgical repair while preserving graft patency.

## Past Medical History

The patient has a history of coronary artery disease, status post 3-vessel coronary artery bypass grafting (CABG) in 2012 (left internal mammary artery to left anterior descending artery, saphenous vein graft [SVG] to the right coronary artery, and SVG-diagonal) with multiple subsequent percutaneous coronary interventions (PCIs). Additional comorbidities included hypertension, class I obesity, hyperlipidemia with intolerance to statins and evolocumab, and gastroesophageal reflux disease secondary to hiatal hernia. The patient is a nonsmoker.

## Differential Diagnosis

The differential diagnosis at this stage included progression of native coronary artery disease, in-stent restenosis, and structural cardiac disease (eg, valvular disease or heart failure).

## Investigations

**Figure 1:**
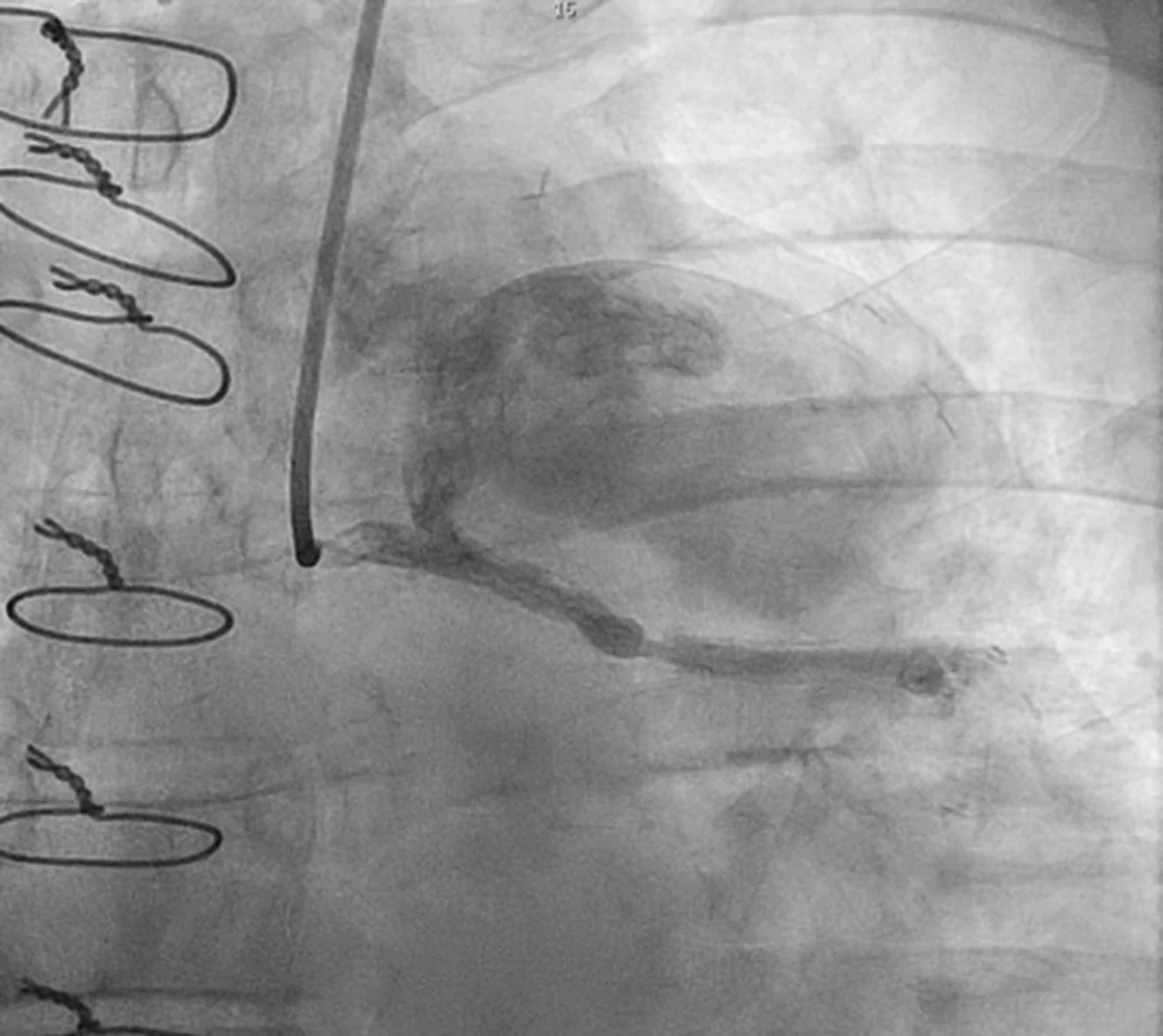
Initial Angiogram Showing a Large Pseudoaneurysm of the Saphenous Vein Graft

Given persistent symptoms, the patient underwent elective coronary angiography that revealed a large mass arising from the proximal segment of the SVG to the diagonal branch with active extravasation of contrast material into the sac (Figure 1). Previously placed stents were also visualized within the proximal and mid segments of the SVG graft.

**Figure 2:**
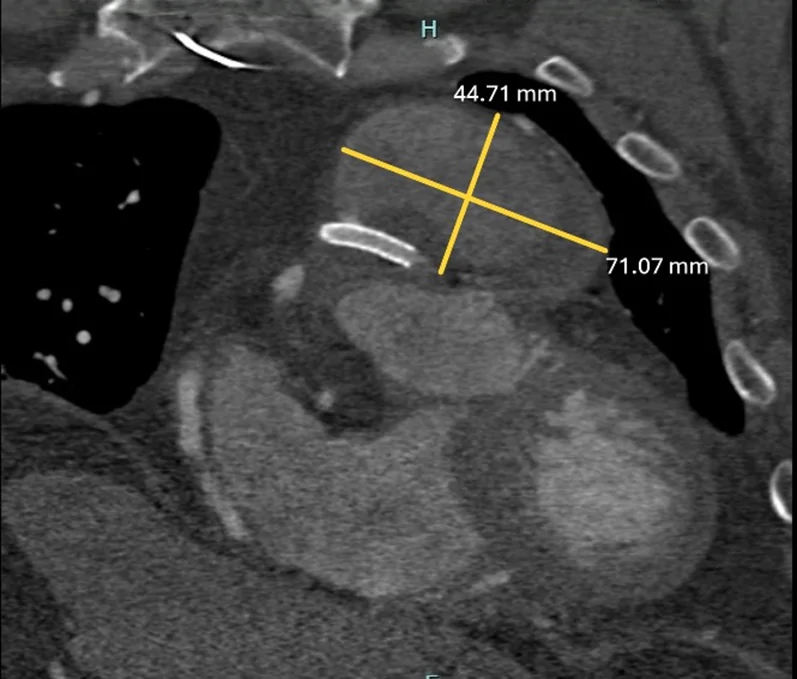
Coronary Computed Tomography Angiogram Shows A Large Pseudoaneurysm of the Saphenous Vein Graft

To further characterize the lesion, coronary computed tomography angiography was performed. Computed tomography angiography demonstrated a 71 × 45-mm saccular pseudoaneurysm with its neck measuring 1.7 mm located cephalad to the pulmonary artery bifurcation and extending superiorly toward the aortic arch within the left anterior mediastinum. The lesion was noted to contain multilayered thrombus with central opacification within the sac radiologically consistent with pseudoaneurysm (Figure 2).

## Management (Medical/Interventions)

Roadmap angiography was performed to delineate the severity and extent of the bypass graft lesion. An 80% stenotic lesion in the mid-segment of the SVG was crossed with a 0.014-inch run-through coronary guidewire. Intravascular ultrasound (IVUS) was performed to define proximal and distal landing zones, demonstrating minimal plaque burden and calcification adjacent to the previously placed proximal SVG stent from which the pseudoaneurysm originated.

The midgraft stenosis was first treated with deployment of a 3.5 × 16-mm Synergy drug-eluting stent. Attention was then directed to the pseudoaneurysm. A self-expanding Gore Viabahn-covered stent was deployed across the neck of the pseudoaneurysm to exclude the lesion. Postdilation was performed using a 5.0 × 15-mm noncompliant Emerge balloon and inflated throughout the covered stent.

Because of persistent residual flow into the pseudoaneurysm neck, an additional 5.0 × 15-mm Papyrus-covered stent was deployed proximally to the Viabahn stent and inflated to 14 atm for 30 seconds. Further postdilation was performed with a 4.5 × 15-mm noncompliant Emerge balloon, inflated to 18 atm for 30 seconds, followed by proximal optimization at 19 atm for 5 seconds.

**Figure 3:**
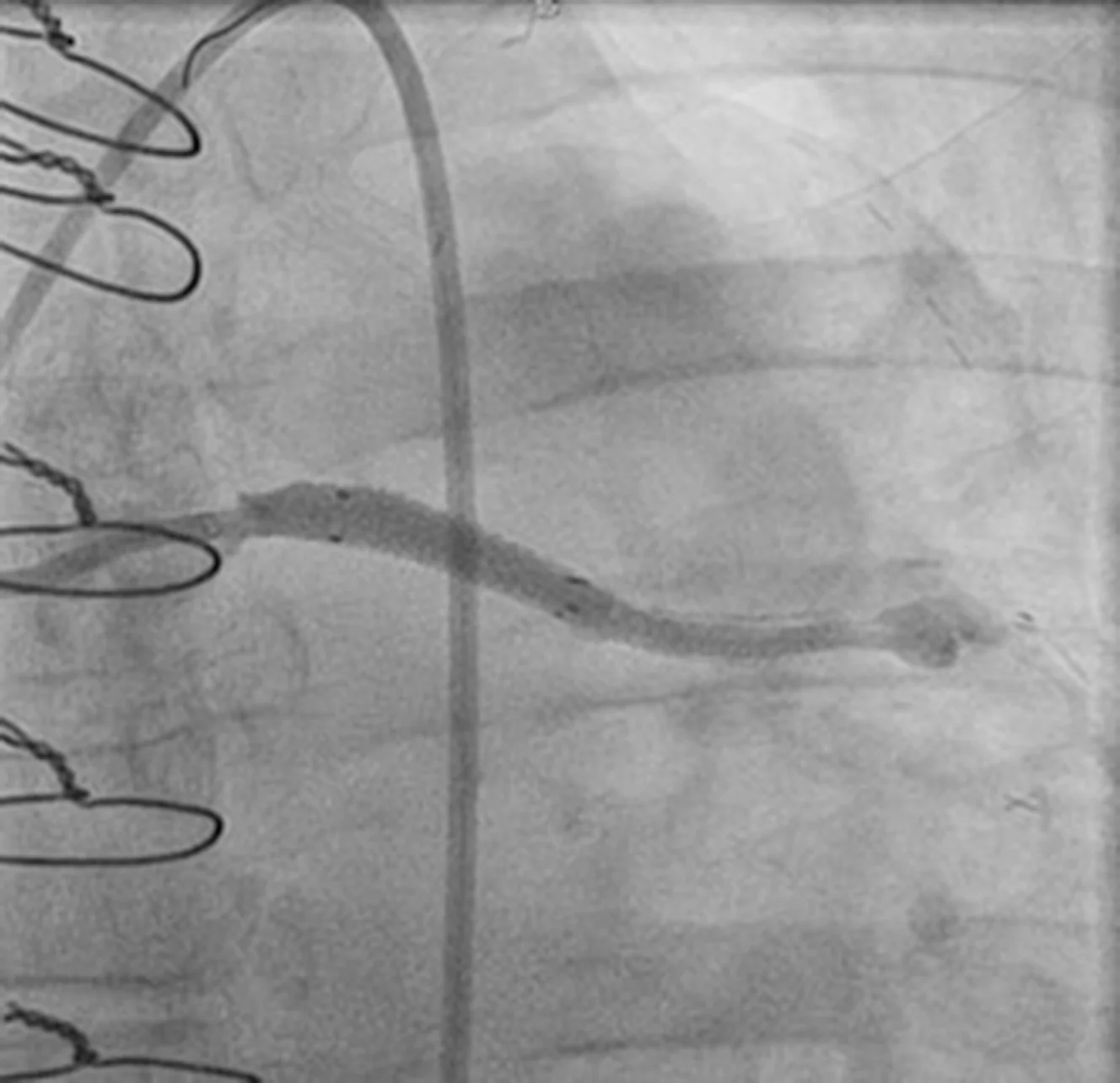
Angiography After Stent Deployment Showing Complete Sealing of the Pseudoaneurysm

Postprocedural IVUS confirmed excellent stent expansion and apposition without procedural complications. Final angiography demonstrated complete exclusion of the pseudoaneurysm with no residual stenosis and preserved TIMI 3 flow throughout the vessel (Figure 3). The patient was advised dual antiplatelet therapy with aspirin and clopidogrel for a minimum of 12 months.

## Outcome and Follow-Up

At a 2-month follow-up, the patient reported marked improvement in dyspnea on exertion, with persistent mild, predominantly nonexertional chest discomfort. To evaluate for complete pseudoaneurysm exclusion, repeat left heart catheterization was performed showing persistent exclusion with no evidence of in-stent restenosis. Given the atypical nature of symptoms and a known hiatal hernia, a noncardiac etiology was suspected, prompting referral to gastroenterology. Ambulatory pH monitoring confirmed severe gastroesophageal reflux, which was determined to be the primary driver of his residual symptoms.

## Discussion

Saphenous vein graft (SVG) aneurysms are defined as focal dilations of a bypass graft measuring at least 1.5 times the diameter of the adjacent reference vessel and represent a rare but potentially life-threatening complication of CABG.

SVG aneurysms are classified as true aneurysms or pseudoaneurysms. True aneurysms involve dilation of all 3 layers of the graft wall and most commonly occur within the body of the conduit as a consequence of progressive degeneration, arterialization, and atherosclerosis.^1^ In contrast, pseudoaneurysms result from focal disruption of 1 or more layers of the graft wall and typically arise at anastomotic sites or areas of previous instrumentation. These lesions may be categorized as early when identified within 12 months of CABG or late when detected more than 5 years after surgery (Table 1).^2^ Early lesions are more often pseudoaneurysms related to technical complications such as suture dehiscence or graft infection, whereas late lesions are more commonly true aneurysms associated with progressive graft degeneration.^2^, ^3^, ^4^, ^5^ However, this temporal distinction is not absolute. Late pseudoaneurysms, although uncommon, have been described in association with previous PCI, stent-related vessel injury, infection, or chronic weakening of a previously disrupted graft segment.^5^ Accordingly, the timing of presentation should be interpreted as one clue among several, whereas definitive classification relies on lesion morphology, location, previous procedural history, and multimodality imaging findings.

**Table 1:** SVG Aneurysms May Present Early or Late Following CABG and Differ in Mechanism, Pathology, and Clinical Characteristics

Feature	Early SVG Aneurysm/Pseudoaneurysm	Late SVG Aneurysm/Pseudoaneurysm
Timing after CABG	<12 mo	>5 y (often 10-15 y)
Most common type	Pseudoaneurysm	True aneurysm
Primary mechanism	Technical or surgical injury	Degenerative changes in graft
Typical location	Anastomotic sites (proximal or distal)	Midgraft body
Key risk factors	Surgical suture line disruption; graft infection; trauma from catheterization or PCI; technical injury during graft harvesting	Progressive graft atherosclerosis; arterialization of the vein graft; hyperlipidemia; hypertension; long-term graft degeneration
Pathology	Vessel wall disruption contained hematoma	Dilatation involving all 3 vessel layers

CABG = coronary artery bypass graft; PCI = percutaneous coronary intervention; SVG = saphenous vein graft.

SVG aneurysms typically present in the sixth decade of life and occur predominantly in men (87% of cases), likely reflecting the higher prevalence of atherosclerotic coronary disease in this population.^6^ Clinical presentation of SVG aneurysms and pseudoaneurysms is highly variable, ranging from incidental findings on imaging to angina, dyspnea, myocardial infarction, rupture, and sudden cardiac death.^7^^,^^8^ In our case, progressive exertional chest discomfort and dyspnea prompted coronary angiography, which identified the lesion.

**Figure 4:**
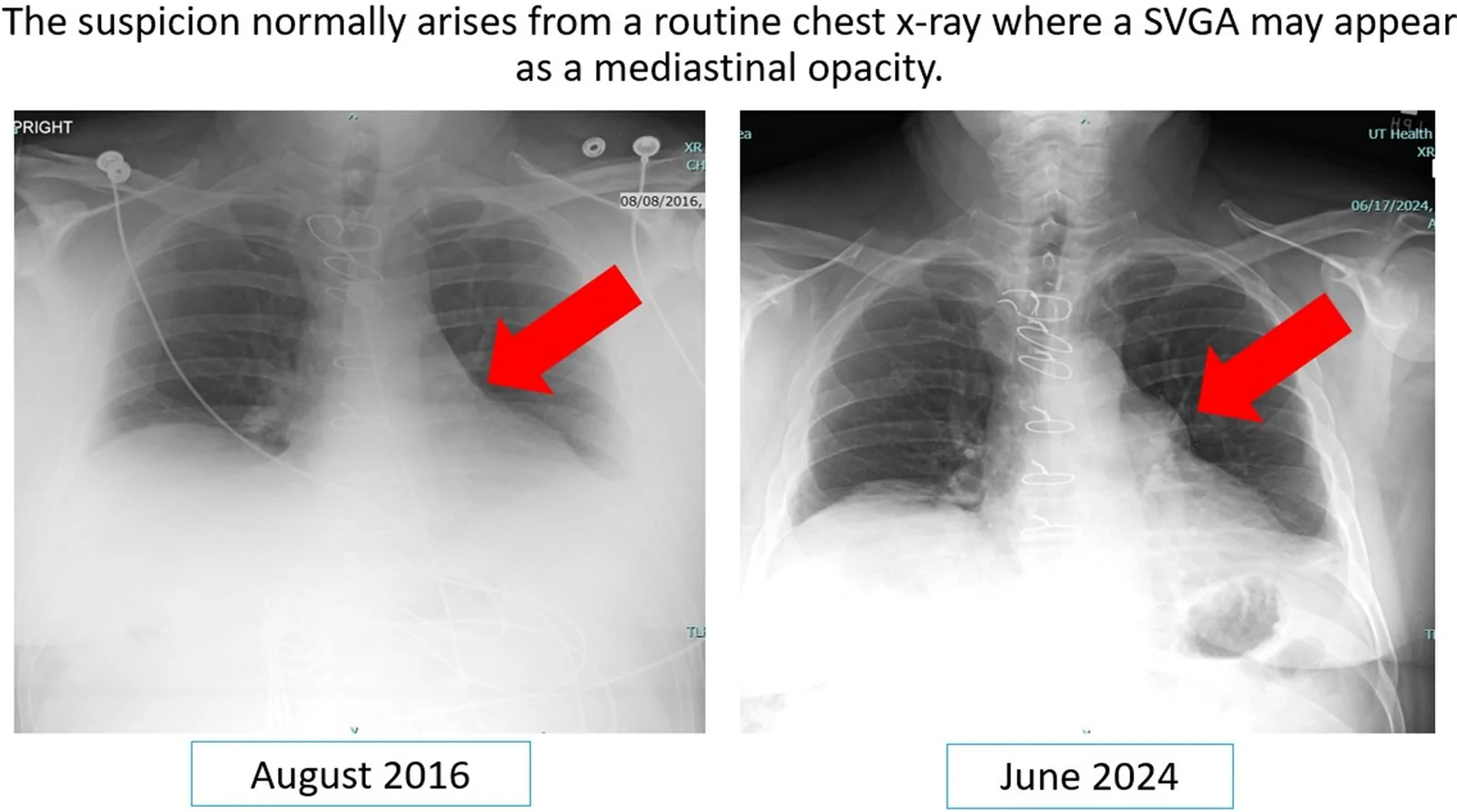
Suspicion of an SVGA May Arise From A Routine Chest X-Ray Where an SVGA May Appear as a Mediastinal Opacity Red arrows demonstrate the interval progression of the mediastinal opacity. SVGA = saphenous vein graft aneurysm.

Multimodality imaging plays a central role in the evaluation of coronary bypass graft aneurysms and may include cardiac magnetic resonance imaging, coronary computed tomography angiography, and invasive coronary angiography with adjunctive intravascular imaging such as IVUS or optical coherence tomography. In some cases, initial recognition may be prompted by a chest radiograph demonstrating a mediastinal mass, as observed in the present case (Figure 4).

In the absence of formal guideline recommendations, management of SVG aneurysms and pseudoaneurysms remains individualized and is guided by lesion morphology, graft patency, distal myocardial viability, and patient-specific surgical risk. Available treatment options include conservative medical therapy, surgical repair (aneurysm resection, graft ligation, or redo CABG), and percutaneous approaches such as covered stent implantation, coil embolization, and vascular plug occlusion.^9^ Surgical repair has historically been considered the definitive treatment but is associated with substantial morbidity because of the technical complexity of repeat sternotomy and the significant comorbidity burden common in this population.^6^^,^^10^ Over the past decade, percutaneous strategies have emerged as an effective less invasive alternative, particularly in patients with high surgical risk or when preservation of graft patency is desired.

In the present case, a multidisciplinary heart team discussion involving cardiothoracic surgery, interventional cardiology, and general cardiology was undertaken. Given the patient's elevated surgical risk, preserved graft patency, and preference to avoid the morbidity and rehabilitation associated with redo sternotomy, percutaneous intervention was favored. This approach is further supported by lower reported mortality with PCI compared with surgical repair (6.1% vs 13.9%).^11^ Coil embolization was considered; however, because of the proximal location of the lesion, the narrow pseudoaneurysm neck, and lack of equipment availability, covered stent implantation was deemed the most appropriate strategy. The patient subsequently underwent IVUS-guided PCI with sequential deployment of covered stents, resulting in complete exclusion of the pseudoaneurysm while preserving graft patency.

Given the limited available data, the optimal antithrombotic regimen and duration following implantation of multiple covered stents within an SVG remain uncertain. This decision is further complicated by the higher rates of restenosis and repeat revascularization reported with covered stents.^12^ Given the patient's previous intolerance to ticagrelor and the prohibitive cost of prasugrel, dual antiplatelet therapy with aspirin and clopidogrel was prescribed for a minimum of 12 months. Beyond 12 months, continuation of antiplatelet therapy will be individualized based on bleeding and ischemic risk.

## Conclusions

SVG pseudoaneurysms are rare but potentially life-threatening late complications of CABG. Their clinical presentation can be variable and may mimic other cardiac or mediastinal pathologies, making multimodality imaging essential for accurate diagnosis and procedural planning. In the absence of dedicated guideline recommendations, management should be individualized based on aneurysm characteristics, graft patency, and patient surgical risk through a heart team approach. This case demonstrates that IVUS-guided percutaneous intervention with covered stent placement can be an effective minimally invasive strategy to successfully exclude a large SVG pseudoaneurysm while preserving graft flow, highlighting the growing role of endovascular therapies in the management of complex graft complications.

## Funding Support and Author Disclosures

The authors have reported that they have no relationships relevant to the contents of this paper to disclose.

## Appendix

Video 1 This Angiogram Shows Filling of the SVG Pseudoaneurysm on Initial Angiography

Video 2 This Angiogram Shows Residual Flow Into the Pseudoaneurysm After the Initial Covered Stent Placement

Video 3 This Angiogram Shows Complete Sealing of the SVG Pseudoaneurysm Neck After the Second Stent Implantation
